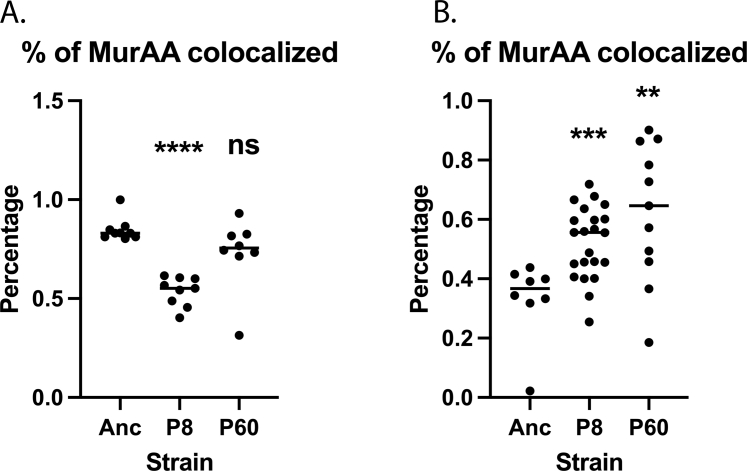# Enolpyruvate transferase MurAA^A149E^, identified during adaptation of *Enterococcus faecium* to daptomycin, increases stability of MurAA–MurG interaction

**DOI:** 10.1016/j.jbc.2023.102912

**Published:** 2023-01-14

**Authors:** Yue Zhou, Budi Utama, Shivendra Pratap, Adeline Supandy, Xinhao Song, Truc T. Tran, Heer H. Mehta, Cesar A. Arias, Yousif Shamoo

**Affiliations:** 1 Department of Biosciences, Rice University, Houston, Texas, USA; 2 Shared Equipment Authority, Rice University, Houston, Texas, USA; 3 Center for Infectious Diseases Research, Houston Methodist Research Institute, Houston, Texas, USA; 4 Division of Infectious Diseases, Houston Methodist Hospital, Houston, Texas, USA

**Keywords:** antibiotic resistance, Enterococcus, UDP-N-acetylglucosamine enolpyruvyl transferase, peptidoglycan, X-ray crystallography, enzyme kinetics, immunofluorescence microscopy, BHI, Brain Heart Infusion, BSA, bovine serum albumin, DAP, daptomycin, FOS, fosfomycin, MIC, minimum inhibitory concentration, MST, microscale thermophoresis, PDB, Protein Data Bank, PEP, phosphoenolpyruvate

## Abstract

Daptomycin (DAP) is an antibiotic frequently used as a drug of last resort against vancomycin-resistant enterococci. One of the major challenges when using DAP against vancomycin-resistant enterococci is the emergence of resistance, which is mediated by the cell-envelope stress system LiaFSR. Indeed, inhibition of LiaFSR signaling has been suggested as a strategy to “resensitize” enterococci to DAP. In the absence of LiaFSR, alternative pathways mediating DAP resistance have been identified, including adaptive mutations in the enolpyruvate transferase MurAA (MurAA^A149E^), which catalyzes the first committed step in peptidoglycan biosynthesis; however, how these mutations confer resistance is unclear. Here, we investigated the biochemical basis for MurAA^A149E^-mediated adaptation to DAP to determine whether such an alternative pathway would undermine the potential efficacy of therapies that target the LiaFSR pathway. We found cells expressing MurAA^A149E^ had increased susceptibility to glycoside hydrolases, consistent with decreased cell wall integrity. Furthermore, structure–function studies of MurAA and MurAA^A149E^ using X-ray crystallography and biochemical analyses indicated only a modest decrease in MurAA^A149E^ activity, but a 16-fold increase in affinity for MurG, which performs the last intracellular step of peptidoglycan synthesis. Exposure to DAP leads to mislocalization of cell division proteins including MurG. In *Bacillus subtilis*, MurAA and MurG colocalize at division septa and, thus, we propose MurAA^A149E^ may contribute to DAP nonsusceptibility by increasing the stability of MurAA–MurG interactions to reduce DAP-induced mislocalization of these essential protein complexes.

The rapid emergence of bacterial antibiotic resistance is a worldwide crisis that threatens to undermine our most effective means to control infectious diseases (1). The Centers for Disease Control and Prevention estimates that antibiotic-resistant bacteria cause an estimated 2.8 million infections and 35,000 deaths each year in the United States. Enterococci are prominent members of the ESKAPE pathogen family and are among the organisms of concern within the clinical community (2). The use of antibiotics to combat enterococcal infections has led to more resistant strains including vancomycin-resistant enterococci that are listed by the Centers for Disease Control and Prevention as a serious threat (2, 3).

Daptomycin (DAP) is a cyclic lipopeptide that is used widely to treat vancomycin-resistant enterococci infections (4, 5). Although the precise mechanism of DAP killing is not fully understood, it is known that DAP inserts into the cell membrane in a calcium-dependent manner, preferably in regions rich in phosphatidylglycerol (6, 7). Recent studies indicate that DAP and Ca^2+^ associate in the cell membrane to form a tripartite complex with phosphatidylglycerol and bactoprenyl-coupled lipid precursors, affecting peptidoglycan synthesis. Indeed, reduced amounts of phosphatidylglycerol can decrease DAP binding to the cell membrane (8, 9). Moreover, DAP binding has pleiotropic effects on the cell membrane including delocalization and displacement of essential proteins involved in cell wall synthesis, such as the lipid II synthase, MurG, further impairing cell wall synthesis, leading to cell death (10).

As resistance to DAP becomes more common among multidrug-resistant gram-positive organisms (*e.g.*, *Enterococcus faecalis*, *Enterococcus faecium*, and methicillin-resistant *Staphylococcus aureus*), new strategies to limit the evolution of resistance and to use DAP in combinatorial therapies with other antibiotics has gained interest (6, 11, 12, 13). In *E. faecalis*, the LiaFSR cell-envelope-stress-response system mediates remodeling of anionic phospholipid microdomains presumably to “divert” DAP from critical septal areas of peptidoglycan synthesis (14, 15, 16, 17). In *E. faecium*, the prevailing mechanism of DAP resistance appears to involve electrostatic “repulsion” of the antibiotic molecule from the cell surface, although the LiaFSR also seems to be the major mediator of this phenotype (18, 19, 20). However, alternative pathways have been described but their mechanisms remain poorly understood (18).

Disruption of the LiaFSR pathway induces hypersensitivity to DAP exposure suggesting that DAP-resistant strains could be resensitized by inhibition of the LiaFSR pathway. To explore non-LiaFSR-mediated resistance, Prater *et al.* (21) used *in vitro* experimental evolution to examine how a clinical *E. faecium* strain (HOU503), in which the LiaFSR system was knocked out by deletion of the gene encoding the response regulator LiaR (503F*ΔliaR*), developed resistance to DAP. In a bioreactor environment, mutations in MurAA, a UDP-N-acetylglucosamine enolpyruvate transferase, which catalyzes the first committed step of peptidoglycan synthesis, were commonly observed. Mutations within *divIVA*, a gene that encodes a membrane-binding protein that is localized to the poles and division septa of cells were also observed. Interestingly, studies in *S. aureus* have suggested that DAP exposure produces delocalization of the peptidoglycan synthesis machinery (8). Here, we examine the potential role of changes in MurAA during DAP exposure using a combination of X-ray crystallography, biochemistry, and microscopy. We found that, while the MurAA change (MurAA^A149E^) had no substantial effects in enzymatic activity or structure, the substitution resulted in increased binding affinity for MurG. The MurG enzyme catalyzes the transfer of GlcNAc of UDP-GlcNAc to the C4 hydroxyl MurNAc in lipid I to produce the lipid-linked β-(1,4) disaccharide known as lipid II. MurG is associated with the inner leaflet of the division septa during exponential growth to carry out the last cytoplasmic step of peptidoglycan synthesis (22, 23). DAP exposure can lead to substantial mislocalization of septal proteins and protein assemblies (7). Our results suggest that adaptive mutations that stabilize the peptidoglycan biosynthesis proteins at the septa may mitigate DAP-induced mislocalization of protein assemblies and thereby restore cellular fitness.

## Results

### MurAA adopts a closed conformation with UDP-GlcNAc and fosfomycin bound at the active site

MurAA catalyzes the first committed step of peptidoglycan synthesis, transferring an enolpyruvate moiety from phosphoenolpyruvate (PEP) to UDP-N-acetylglucosamine to produce UDP-GlcNAc-enolpyruvate and inorganic phosphate (24). MurAA comprises two domains with the active site Cys119 located in a flexible loop (Ala114–Ile126) that is solvent exposed in the ligand-free state (25, 26, 27). Previous studies have shown that, in the absence of ligand, MurAA remains in an open state but that, upon ligand binding, the surface-exposed loop undergoes a large conformational change to form a closed conformation (28, 29, 30).

**Figure 1:**
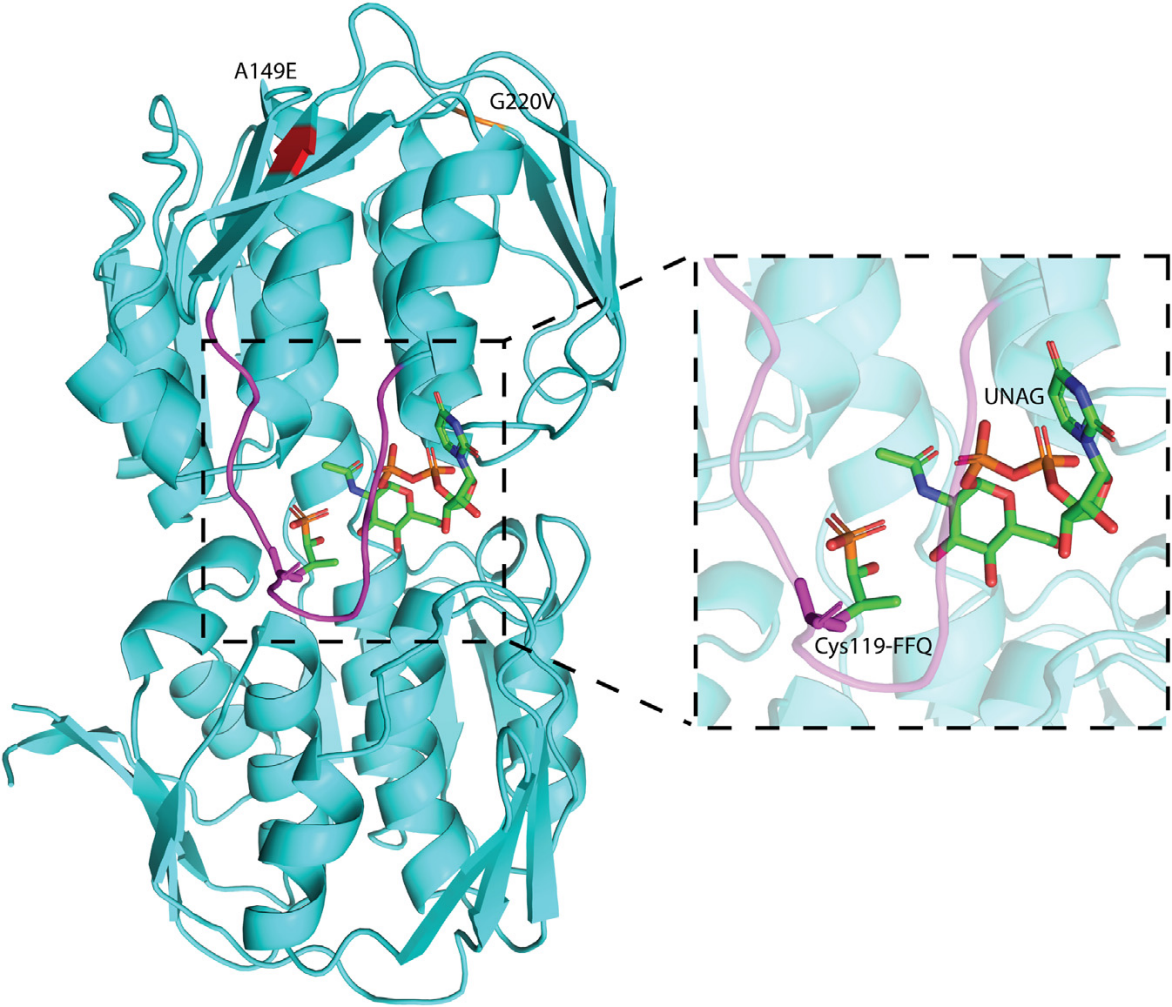
***E. faecium* MurAA adopts a closed conformation with FOS covalently attached to Cys119.** Structural overview of the full-length *E. faecium* MurAA cocrystallized with FOS and UDP-GlcNAc. MurAA is made up of two globular domains connected by two polypeptide linkers. FOS, the inhibitor of MurAA and analogue of phosphoenolpyruvate, covalently binds within the active site at Cys119. UDP-GlcNAc is bound proximal to the interface of the two domains. MurAA is shown in *cyan*, the active site loop (Ala114-Ile126) is in *magenta*. The A149E mutation is shown in *red*. A less common mutation G220V is also indicated in *orange*. FOS and UDP-GlcNAc are shown as *sticks*. FOS, Fosfomycin.

**Supplemental Figure S1:**
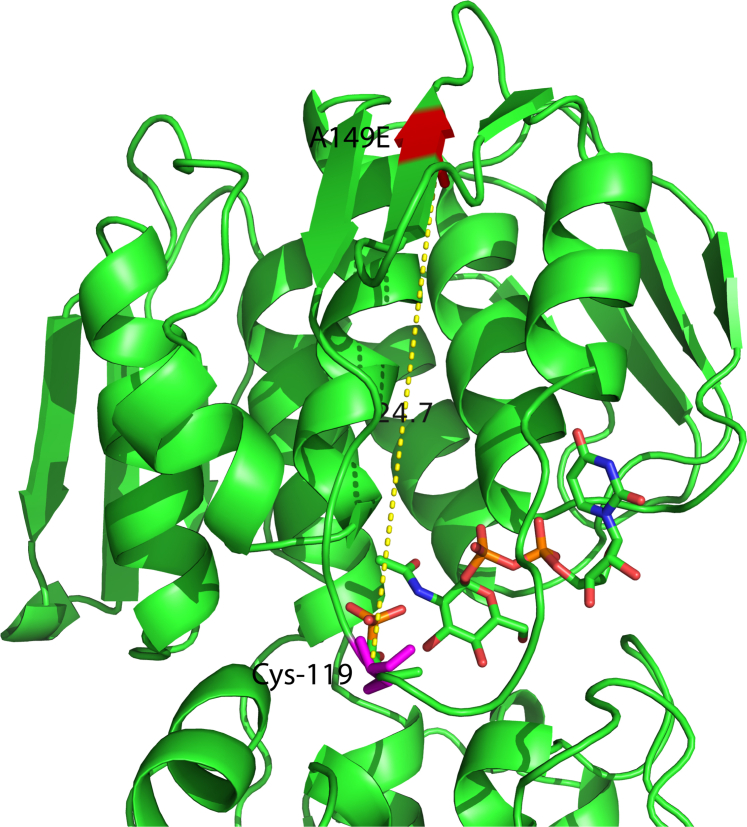


We determined the structure of wildtype *E. faecium* MurAA in complex with its inhibitor, fosfomycin (FOS) (a PEP analogue), and the substrate UDP-GlcNAc at a resolution of 1.65 Å (Protein Data Bank [PDB] id: 7TB0) (Table S1; Figs. 1 and S6). The structure of the MurAA–FOS–UDP-GlcNAc complex was found to be in the closed conformation with UDP-GlcNAc binding at the interface of the two globular domains and FOS covalently attached to the active site Cys119, which is part of the flexible loop (Ala114–Ile126) (magenta in Fig. 1). We refer to the domain containing the active site loop as the “catalytic domain” (residues 21–232) and the other as the “C-terminal domain” (residues 1–20, 233–419). As shown in Figure 1, FOS inhibits MurAA by blocking access to the catalytic site making Cys119 inaccessible to PEP. The adaptive mutation at position 149 is at the surface of the catalytic domain and 24.7 Å away from Cys119 (Fig. S1).

**Figure 2:**
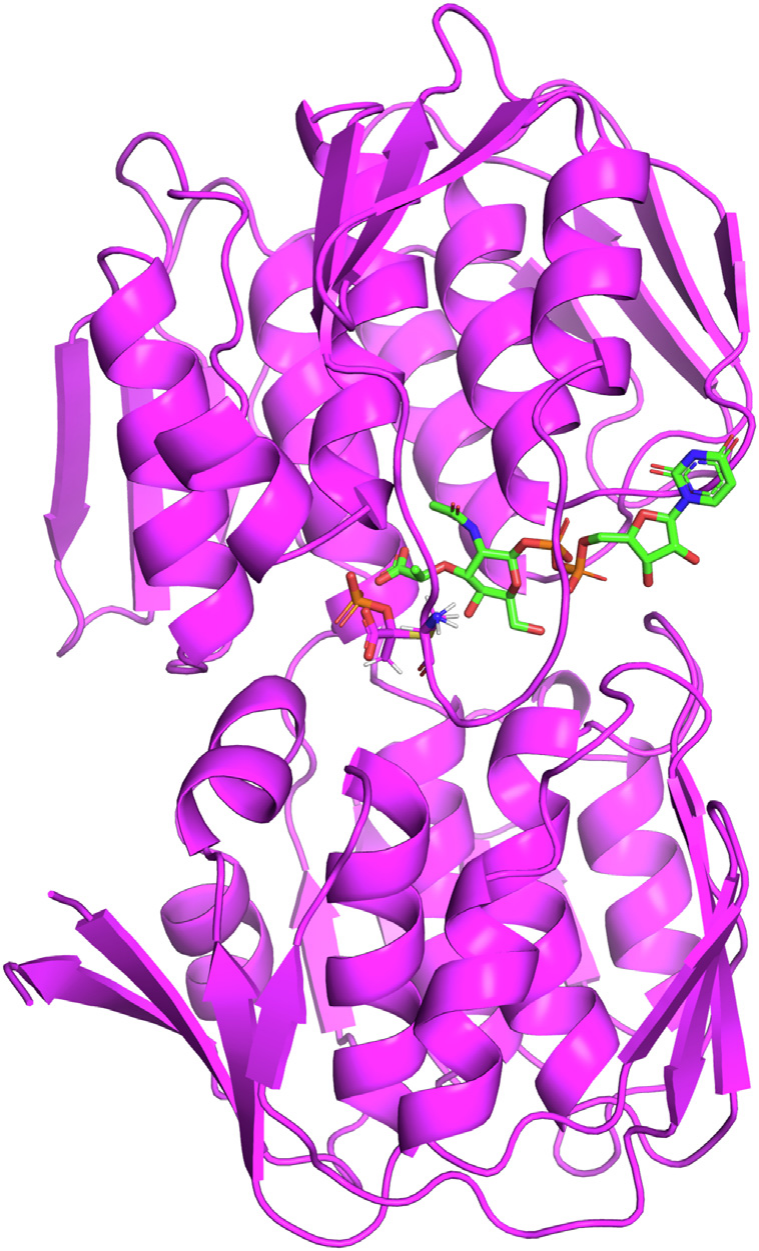
***E. faecium* MurAA adopts a half-open conformation with PEP covalently attached to Cys119.** Structural overview of the full-length *E. faecium* MurAA. PEP covalently binds within the active site at Cys119. UDP-MurNAc is bound proximal to the interface of the two domains. MurAA is shown in *magenta*. PEP and UDP-MurNAc are shown as *sticks*. PEP, phosphoenolpyruvate.

**Figure 3:**
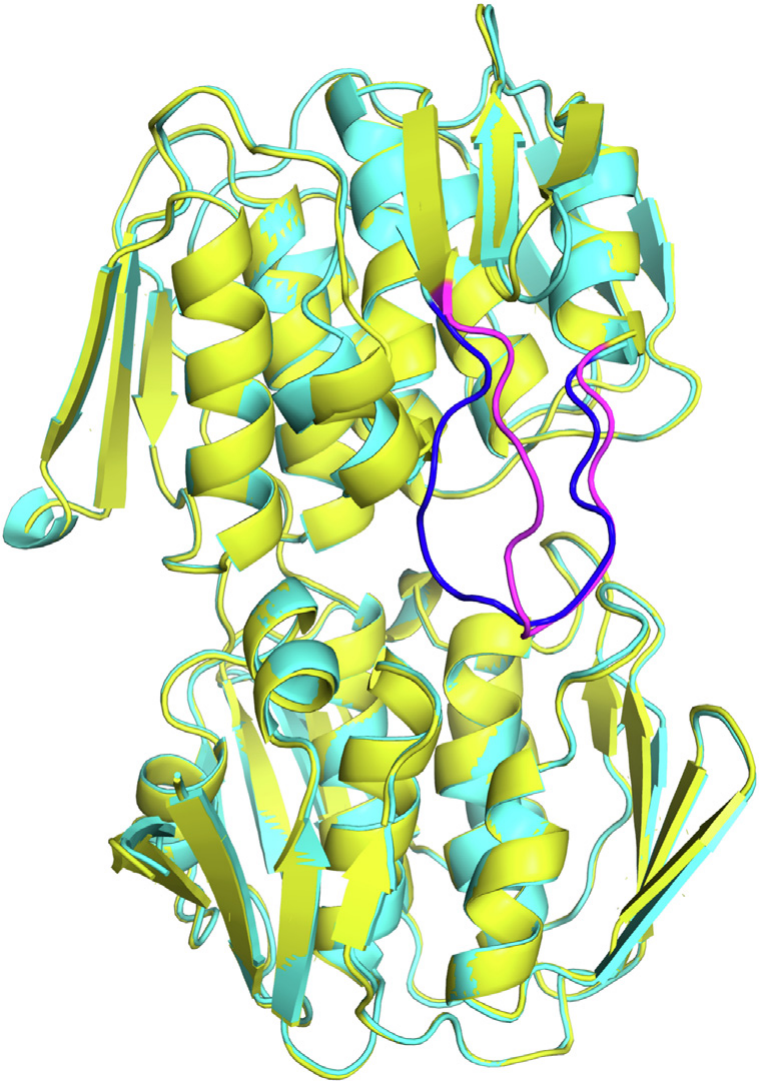
**Superimposition of the closed *E. faecium* MurAA–FOS–UDP-GlcNAc ternary complex and half-open complex of MurAA–PEP–UDP–MurNAc.** The overall structure of MurAA–FOS–UDP-GlcNAc complex (*cyan*) and MurAA–PEP–UDP–MurNAc complex (*yellow*) are similar (rmsd = 0.59 Å), except for the active site loop with an rmsd = 2.68 Å (*blue* for MurAA–FOS–UDP-GlcNAc complex and *magenta* for MurAA–PEP–UDP-MurNAc complex). MurAA is in the closed conformation bound to FOS and UDP-GlcNAc and the half-open conformation in the complex with PEP and UDP-MurNAc. FOS, fosfomycin; PEP, phosphoenolpyruvate.

In order to determine the structure of apo-MurAA, we also attempted to crystallize MurAA without added ligands or cofactors during purification and crystallization. Data were collected up to a resolution of 2.65 Å. Upon inspection of the electron density, we observed significant positive difference density near the thiol group of the active site cysteine (Cys119) and UDP-binding site at the interface of the two domains (Figs. 2 and S6). This was surprising as we did not add any of the ligands during purification and crystallization. A previous study by Zhu *et al.* (30) found that when *Enterobacter cloacae* MurAA was overexpressed in *Escherichia coli* in the absence of any added ligands and without preincubation with phosphate buffer, the enzyme existed in a tight complex with UDP-N-acetylmuramic acid (the product of MurB catalytic reaction with PEP) covalently bound to Cys119 and adopted a half-open conformation. They suggested that UDP–MurNAc and PEP could be bound during the expression in *E. coli*. They also refined previously deposited apo MurA structures and showed that additional density for Cys-PEP adduct was potentially misinterpreted as phosphate and that the “apo-structure” actually contained UDP–MurNAc. The proposed MurAA–UDP–MurNAc complex was referred to as a “dormant complex” (30). Based on these observations, we modeled the covalent modification at the active site cysteine as a Cys119-PEP adduct and UDP–MurNAc at the interface of two domains in *E. faecium* MurAA to form the dormant complex (30). The density for Cys-PEP adducts in all chains was well defined and unambiguous, but the electron density for UDP–MurNAc was found to be weaker and more variable across the individual copies of the asymmetric unit. The observed weaker electron density could be due to a lower occupancy of UDP–MurNAc and may be indicative of partial occupancy. Nonetheless, it was clear that the MurAA–PEP–UDP–MurNAc structure (PDB id: 8D84) (Table S2) displayed a different conformation compared with the MurAA–FOS–UDP-GlcNAc complex and adopted a half-open conformation (Fig. 3). Except for the position of the surface loop (Ala114–Ile126 with rmsd = 2.7 Å) the overall structures of MurAA are quite similar (C-alpha rmsd of 420 residues = 0.6 Å). The structures of *E. faecium* MurAA with FOS–UDP-GlcNAc and by serendipity UDP–MurNAc suggest that, like other members of the MurA family, the protein transitions from an open to closed conformation upon UDP-GlcNAc binding. In our previous study, we also identified a less common G220V substitution during the development of DAP resistance in *E. faecium* HOU515F*ΔliaR* (21). Importantly, positions 149 and 220 are proximal to each other at the protein surface but distal to the active site consistent with a potential role in protein–protein interactions.

### DAP-resistant strains harboring the MurAA^A149E^ mutation have increased sensitivity to mutanolysin and lysozyme

**Figure 4:**
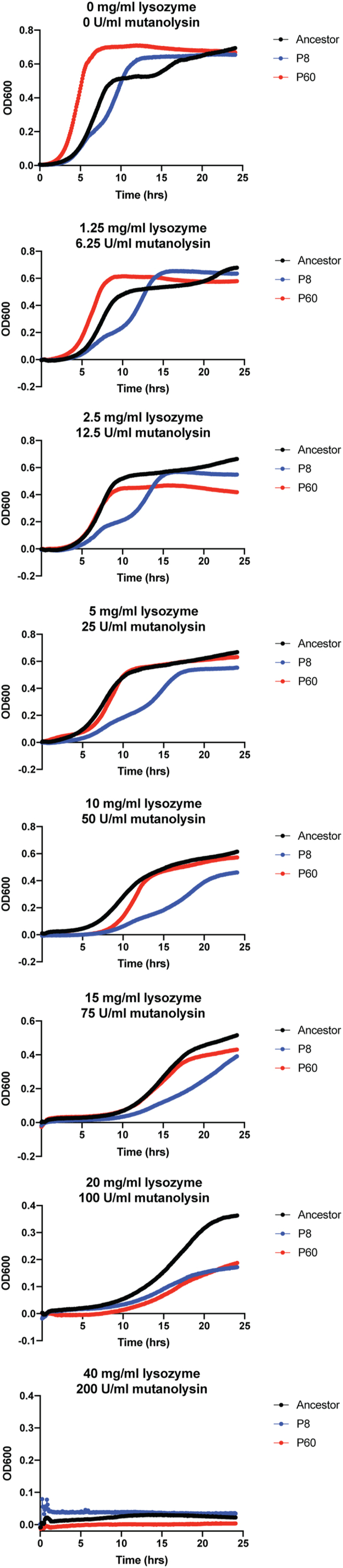
**Daptomycin-resistant strains with the MurAA**^**A149E**^**mutation are more sensitive to mutanolysin and lysozyme treatment.** The growth of *E. faecium* HOU503F_ΔliaR and two bioreactor-evolved end-point isolates containing MurAA^A149E^ (P8 and P60) was measured during treatment with mutanolysin and lysozyme to assess cell wall integrity.

Since MurAA is involved in peptidoglycan biosynthesis, we investigated the effect of the *E. faecium* MurAA^A149E^ substitution on cell wall integrity by measuring the sensitivity of the cells to mutanolysin and lysozyme stress. Cells were grown in BHI growth medium supplemented with two cell wall hydrolases, mutanolysin and lysozyme. Growth of DAP-sensitive HOU503F_*ΔliaR* and its two DAP-resistant derivatives, P8 and P60, with the adaptive mutation MurAA^A149E^, was measured over 24 h. Of note, P8 and P60 were obtained from an earlier study involving experimental evolution of HOU503F_*ΔliaR* to DAP. These isolates had, in addition to *murAA*^A149E^, other mutations (P8: *murAA*^A149E^, *cls*^A20D^, *entfae_809*^A70E^, *entfae_64*^Y83^∗ and P60: *murAA*^A149E^, *cls*^N13I^, *entfae_126*^V30^∗), none of which are known to have a role in peptidoglycan synthesis (21). The minimum inhibitory concentration (MIC) of P8 and P60 to DAP is 8 mg/l in BHI supplemented with 50 mg/l calcium chloride. Figure 4 shows that the lag phases of P8 and P60 were significantly longer compared with that of the ancestor in the presence of increasing concentrations of the glycoside hydrolases. Although other mutations in the *E. faecium* isolates P8 and P60 make it difficult to directly implicate *murAA*^A149E^ in the phenotype, our results suggest that strains harboring the MurAA^A149E^ substitution have cell walls with decreased integrity and take a longer time to establish growth under stress imposed by cell wall hydrolases.

### MurAA^A149E^ has a modestly lower catalytic activity compared with MurAA *in vitro*

**Figure 5:**
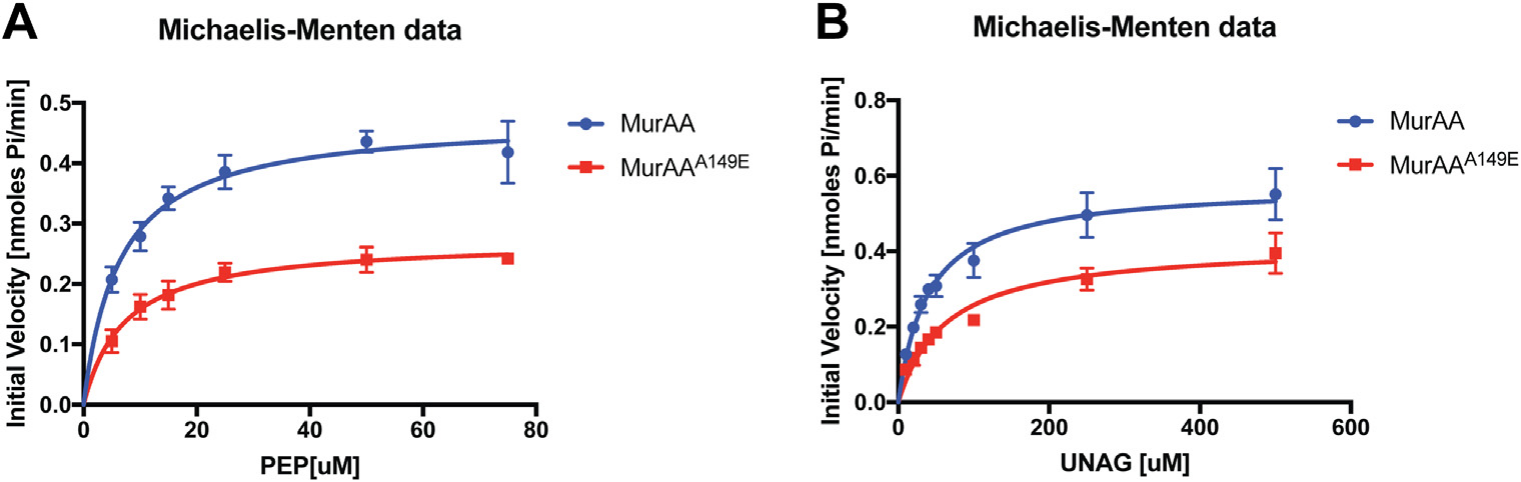
***E. faecium* MurAA**^**A149E**^**has slightly lower activity than MurAA.** Steady-state kinetic analysis of *E. faecium* MurAA (*blue*)/MurAA^A149E^ (*red*). *A*, MurAA activity measured against PEP. The reaction mixture contained 35 nM MurAA, 4 mM UDP-GlcNAc, and an increasing concentration of PEP (from 5 μM to 75 μM). *B*, MurAA activity measured against UDP-GlcNAc. The reaction mixture contained 50 nM MurAA, 1 mM PEP, and an increasing concentration of UDP-GlcNAc (from 10 μM to 500 μM). PEP, phosphoenolpyruvate.

**Supplemental Figure S2:**
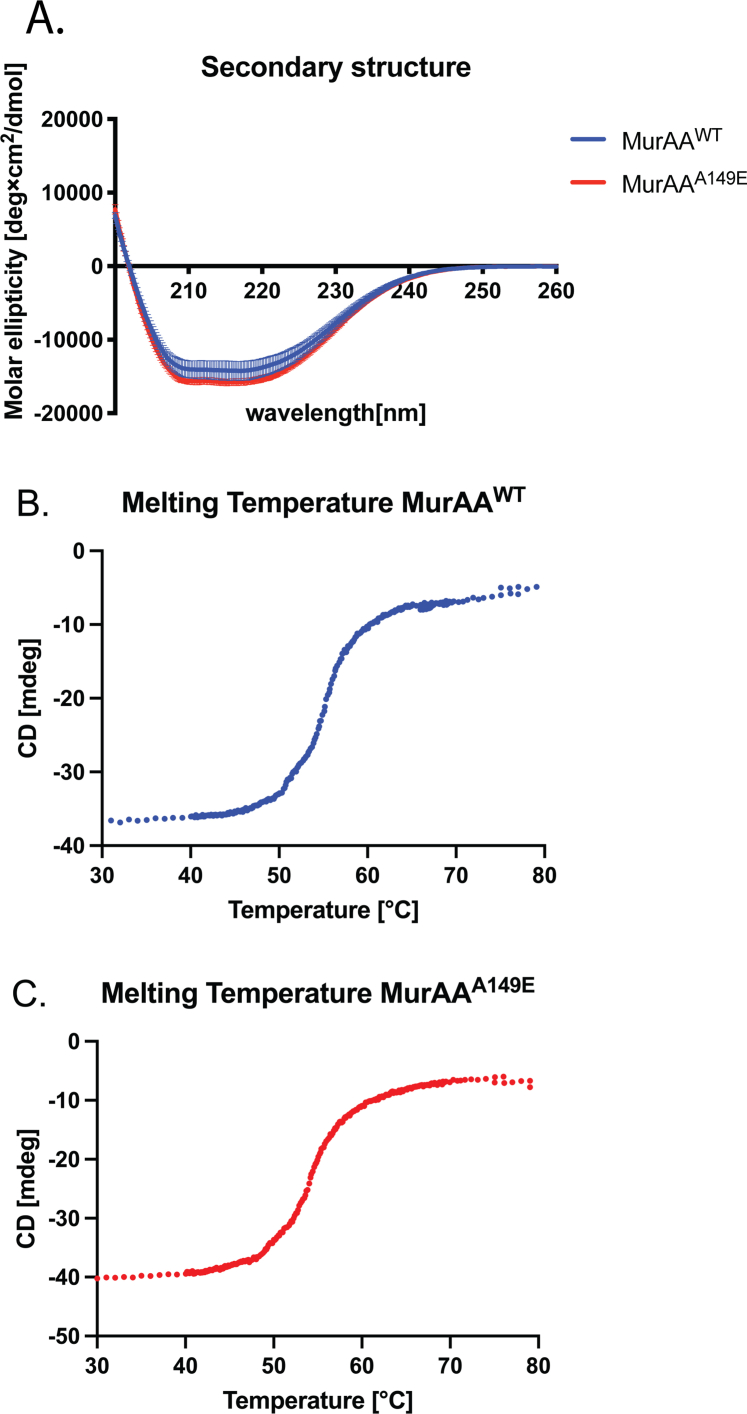


**Supplemental Figure S3:**
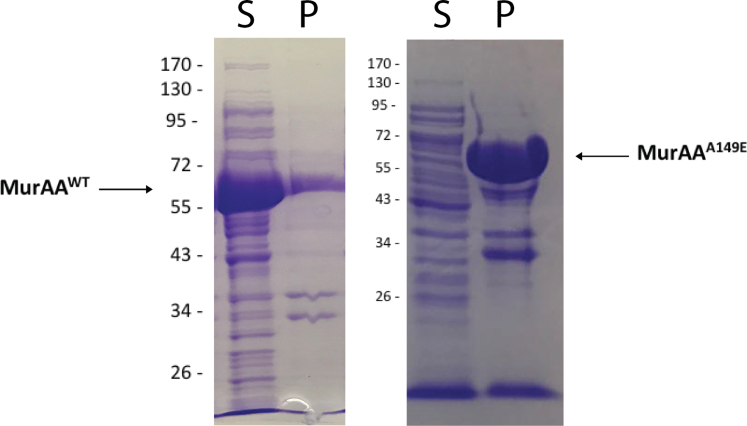


We were also interested in the effect of the A149E mutation on MurAA catalytic properties as even distant mutations can have substantial effects on protein dynamics leading to altered activity or substrate binding. We purified the wildtype and mutant proteins and conducted steady-state enzymatic assays to directly investigate their activities by measuring the Michaelis–Menten reaction rate constants for the two substrates of MurAA, PEP, and UDP-GlcNAc (Table 1 and Fig. 5). While the A149E mutation strongly decreases the solubility of MurAA, circular dichroism (CD) studies suggested that the Ala to Glu substitution in MurAA at position 149 did not produce significant secondary-structure changes (Figs. S2 and S3). MurAA^A149E^ and MurAA have comparable *K*ms but an ∼1.7-fold decrease in *k*_cat_ for PEP compared with the wildtype protein. For the UDP-GlcNAc substrate, MurAA^A149E^ exhibits a higher *K*m value and similar *k*_cat_ to the wildtype enzyme. In general, MurAA^A149E^ shows a lower catalytic activity compared with MurAA. In addition, the *K*m of PEP is ∼6- to 8-fold lower than that of UDP-GlcNAc, which was consistent with studies performed in other organisms (31, 32, 33, 34, 35). Of note, the *K*m values of *E. faecium* MurAA for PEP and UDP-GlcNAc substrates are relatively lower when compared with that of other MurAAs (31, 32, 33, 34, 35).

**Table 1:** Steady-state kinetics measurements of MurAA^WT^/MurAA^A149E^ for phosphoenolpyruvate (PEP) and UNAG

	Vmax for PEP [nmol·min^−1^ mg^−1^]	Km for PEP [μM]	kcat for PEP [s^−1^]	Vmax for UNAG [nmol·min^−1^ mg^−1^]	Km for UNAG [μM]	kcat for UNAG [s^−1^]
MurAA	2264.67 ± 77.27	6.35 ± 0.88	1.81 ± 0.06	1921.91 ± 68.40	39.81 ± 4.35	1.53 ± 0.06
MurAA^A149E^	1302.17 ± 49.08	7.26 ± 1.05	1.04 ± 0.04	1400.71 ± 66.11	63.18 ± 8.25	1.12 ± 0.05

MurAA^A149E^ and MurAA have comparable Kms but an ∼1.7-fold decrease in kcat for PEP compared with the wildtype protein. For the UDP-GlcNAc substrate, MurAA^A149E^ exhibits a higher Km value and similar kcat to the wildtype enzyme.

### MurAA^A149E^ has a reduced reactivity for fosfomycin

**Figure 6:**
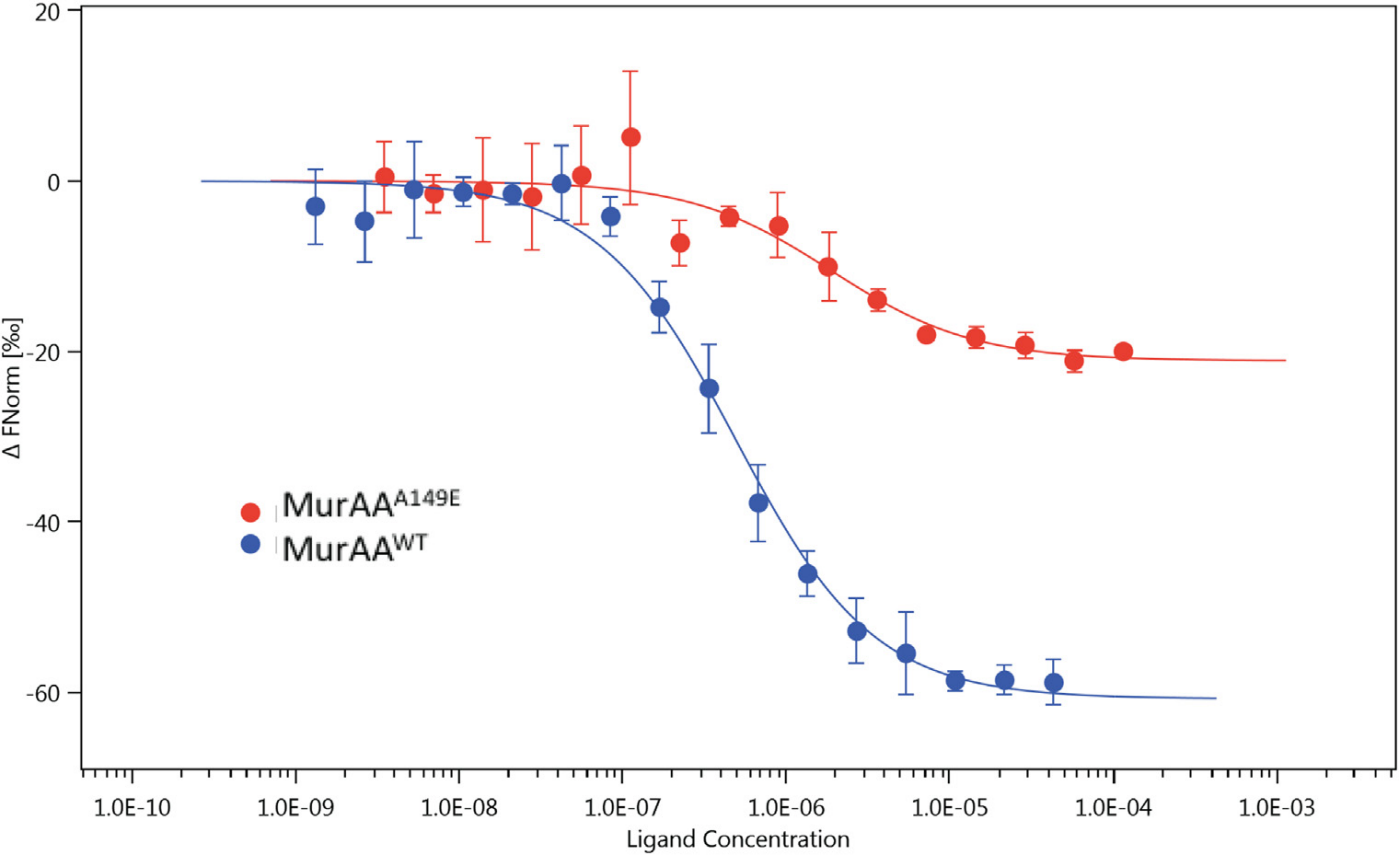
**Mutation A149E decreases MurAA reactivity for fosfomycin (FOS).** Microscale thermophoresis (MST) was used to measure the reactivity of *E. faecium* full-length MurAA binding to its inhibitor, FOS. Fluorescently labeled MurAA^WT^ (*blue*)/MurAA^A149E^ (*red*), 50 nM, and 2 mM UDP-GlcNAc were mixed with an increasing concentration of FOS (from 43.3 μM to 1.32 nM and 115 μM to 3.51 nM, respectively). Mutation A149E decreases MurAA reactivity for FOS by ∼4-fold (*p* value is 0.0184). The ΔFnorm values are calculated from the ratio of fluorescence prior to MST activation/fluorescence after MST activation with consideration of amplitude size and direction.

Fosfomycin is an irreversible inhibitor of MurAA and is structurally analogous to the substrate PEP. FOS covalently binds to the active site Cys119 as shown in the MurAA crystal structure and by previous studies in other organisms (27, 36). We used microscale thermophoresis (MST) to measure the reactivity of MurAA and MurAA^A149E^ for FOS in the presence of UDP-GlcNAc. The concentration of FOS to give a 50% decrease in fluorescence signal was 0.47 ± 0.06 μM for MurAA^A149E^ *versus* 1.87 ± 0.67 μM for wildtype (Fig. 6). The modest 4-fold decrease in FOS reactivity measured using MST for MurAA^A149E^ was consistent with the steady-state enzymatic assay results showing that MurAA^A149E^ had an increased Km for PEP.

### DAP-resistant strains that include the adaptive MurAA^A149E^ mutation have upregulated MurAA and MurAB transcript levels

**Figure 7:**
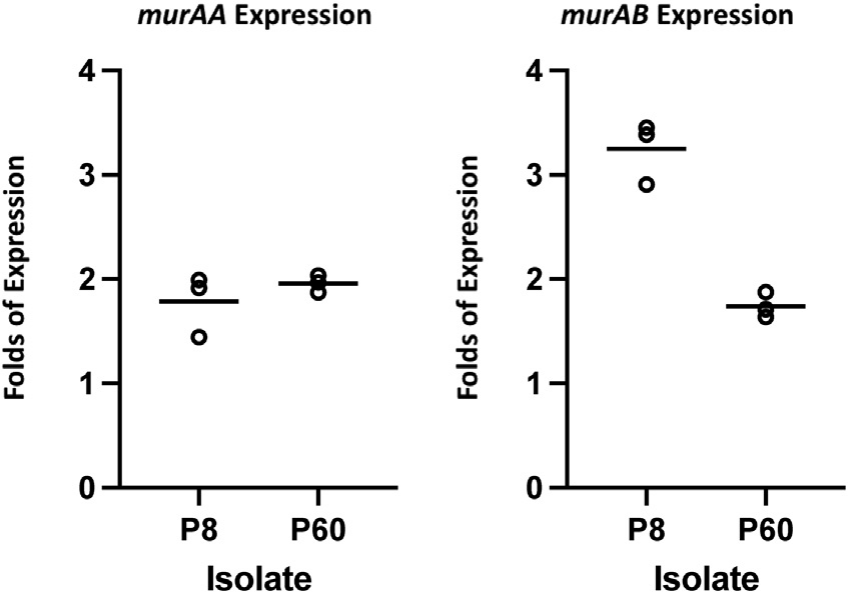
**Daptomycin-resistant strains containing MurAA**^**A149E**^**mutation have increased *murAA* and *murAB* transcript levels compared with the *E. faecium* HOU503F_Δ*liaR* (ancestor).** Reverse transcription–quantitative polymerase chain reaction was used to measure the transcription level of the ancestor and bioreactor-evolved end-point isolates with glucose-1-dehydrogenase 4 (*gdhIV*) as the internal control. P8 and P60 are two daptomycin-resistant strains evolved from the bioreactor environment, sharing the common MurAA^A149E^ mutation in addition to several other mutations. Mutations identified in P8: *murAA*^*A149E*^, *cls*^*A20D*^, *caps*^*A70E*^, *entfae_64*^*Y83*^*∗*. Mutations identified in P60: *murAA*^*A149E*^, *cls*^*N13I*^, *entfae_126*^*V30*^*∗*, *repA plasmid* 1(+214).

Unlike in gram-negative bacteria where *murA* is an essential single-copy gene, *murAA* in low-GC-content gram-positive bacteria is redundant as these organisms have an additional gene that encodes MurAB, an isozyme of MurAA. In *S. aureus*, *murAB* is upregulated when *murAA* is inhibited (33). Since *E. faecium* MurAA^A149E^ activity was modestly reduced compared with wildtype, we investigated whether transcripts of *murAB* would be upregulated and compensate for the potential decreases in MurAA activity. To test this hypothesis, we performed reverse transcription–quantitative polymerase chain reaction (RT-qPCR) to measure the transcript levels of *murAA* and *murAB* in the DAP-sensitive parental and the two DAP-resistant end-point isolates, P8 and P60, that harbor the MurAA^A149E^ substitution. *E. faecium murAA* and *murAB* share 43% identity in sequence and are not located in the same operon. As shown in Figure 7, we found a 2-fold increase in *murAA* transcripts in both *E. faecium* P8 and P60. Moreover, we identified an ∼3-fold and 2-fold increase in *murAB* transcripts for P8 and P60, respectively. Taken together, our results suggest that an increase of transcription of *murAA* and *murAB* may help to compensate for the modest activity loss of MurAA^A149E^.

### The MurAA^A149E^ mutant has a markedly increased affinity for MurG compared with wildtype MurAA

**Figure 8:**
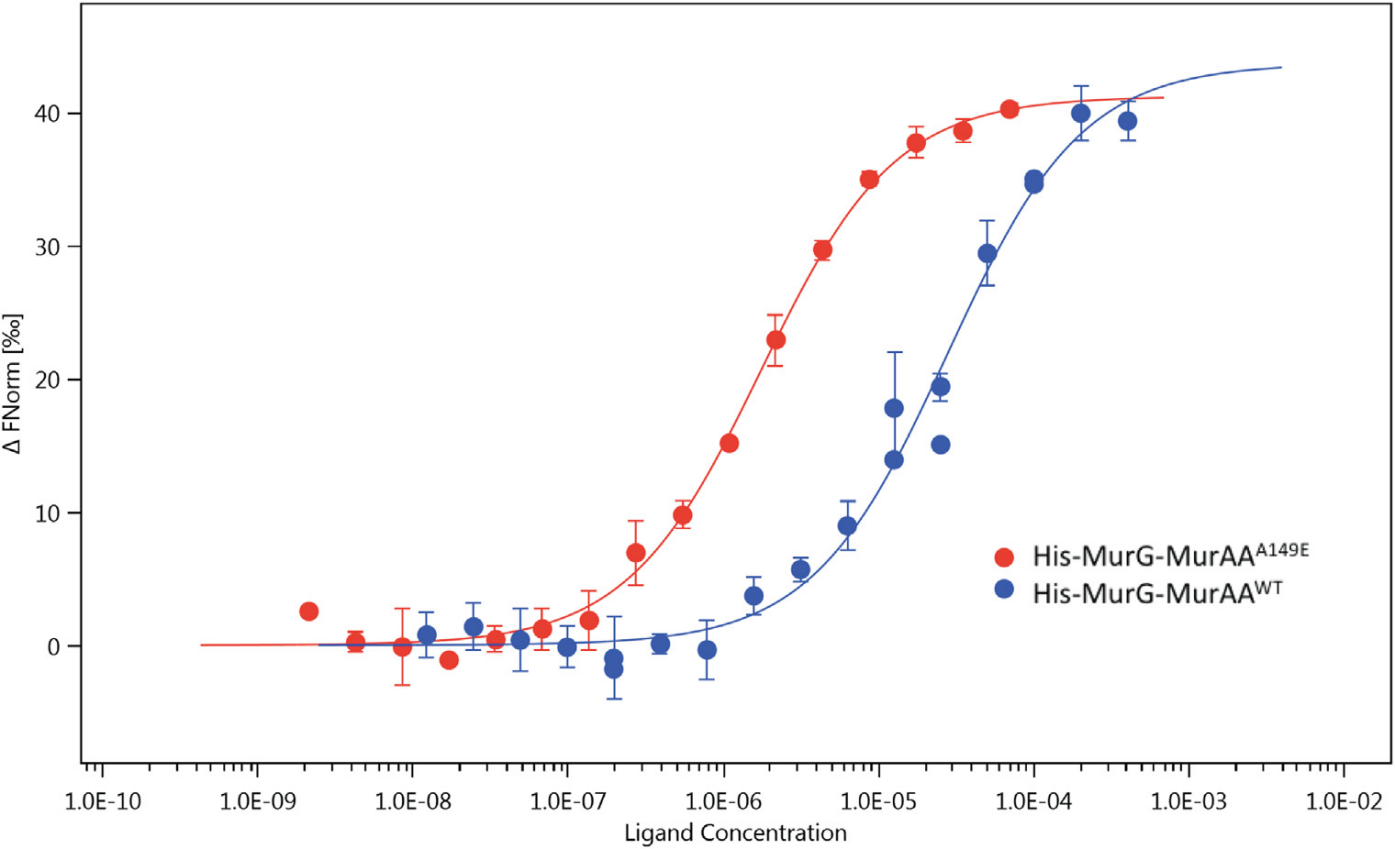
**A149E mutation in MurAA increased its affinity to MurG.** Microscale thermophoresis was used to measure MurG binding to MurAA^WT^ and MurAA^A149E^. Increasing concentrations of MurAA (*blue*) or MurAA^A149E^ (*red*) were added to 50 nM of fluorescently labeled MurG in a buffer containing 20 mM Hepes pH 7.5, 150 mM NaCl, and 0.05% Tween. MurAA has a k_d_ value of 27.68 ± 4.00 μM, and MurAA^A149E^ has a k_d_ value of 1.72 ± 0.13 μM.

**Supplemental Figure S4:**
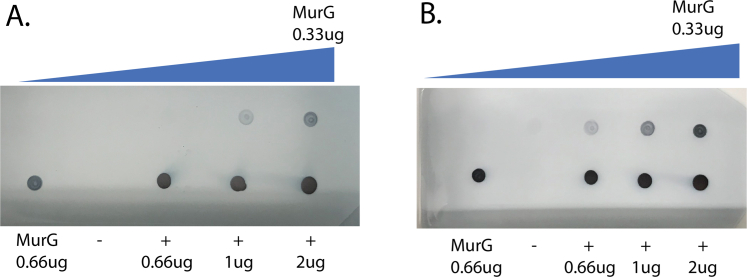


Our data suggested that the MurAA^A149E^ substitutions did not substantially affect the enzymatic activity compared with wildtype MurAA and that the A149E mutation was positioned on the protein surface, away from the active site. Thus, we postulated that the A149E substitution in MurAA may have altered potential interactions with other proteins involved in peptidoglycan synthesis. In *Bacillus subtilis*, studies have shown that MurA and MurG, the first and last enzymes of the peptidoglycan synthesis pathway within the cytoplasm, colocalize at the division septa, lateral walls, and poles during the exponential phase (37). It has been proposed that spatial localization to the septa brings the MurA–MurG reaction centers into closer proximity to facilitate efficient peptidoglycan synthesis (37). Interestingly, MurG has been shown to be displaced from the inner membrane leaflet upon DAP exposure (10). Therefore, we investigated whether MurAA^A149E^ exhibited a change in affinity for MurG compared with wildtype MurAA using MST and dot blot assays. We observed a marked change in affinity for MurG in the MurAA^A149E^ enzyme. Indeed, dissociation constants measured by MST for the MurAA–MurG complex were 27.68 ± 4.00 μM and 1.72 ± 0.13 μM for MurAA^A149E^-MurG (Fig. 8). Our data suggested that mutation of Ala to Glu at position 149 increases the affinity of MurAA for MurG by ∼16-fold. We further confirmed this interaction using a dot blot assay (Fig. S4). The concentration-dependent increase in affinity of MurAA^A149E^ for MurG determined using the dot blot assay was in good agreement with the increase in affinity measured by MST.

### MurAA^A149E^ partially restored DAP-induced MurAA delocalization

**Figure 9:**
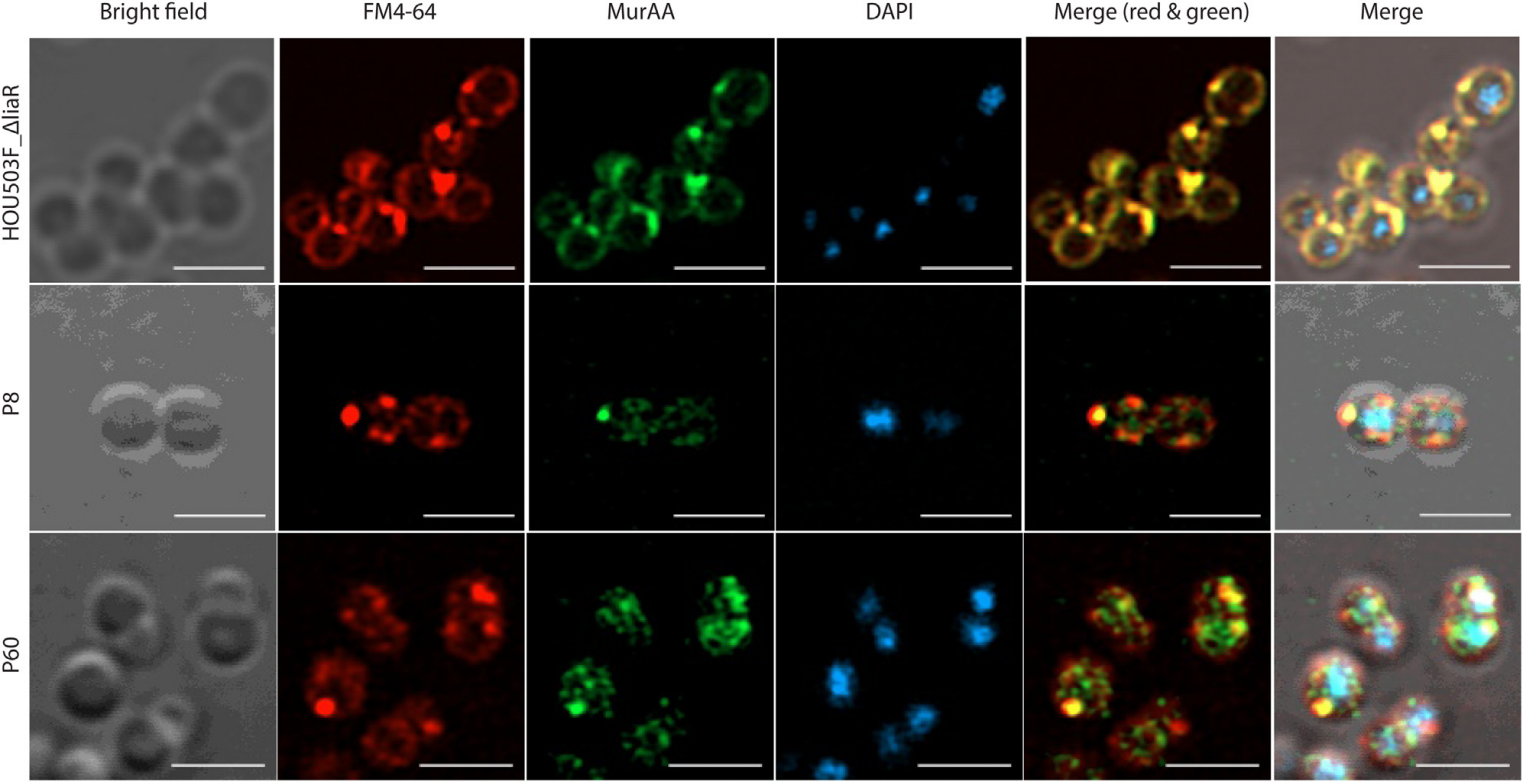
**MurAA principally localizes at the septa and poles.** Representative micrographs of *E. faecium* HOU503F_Δ*liaR* (ancestor) and the two *murAA*^A149E^ isolates P8 and P60. Mid-log phase cells in the absence of daptomycin were harvested, fixed, permeabilized, and stained with membrane dye FM 4-64 FX (*red*), DNA dye DAPI (*blue*), and MurAA antibody (*green*). *Top row* (HOU503F_Δ*liaR*): *Yellow* in merged images indicates colocalization of membrane dye FM 4-64 FX and MurAA protein. *Middle* and *bottom rows* (P8 and P60): MurAA^A149E^ was distributed in the membrane and cytosol. The membrane-associated portion still showed a strong localization of MurAA at the septa and poles. In P8 and P60, FM 4-64 FX shows a speckled distribution pattern. Both P8 and P60 were much more sensitive to mutanolysin/lysozyme digestion during permeabilization, which was consistent with a significantly weakened cell wall. The scale bar represents 2 μm.

As MurAA^A149E^ stabilized the MurAA–MurG complex *in vitro*, we went on to study whether the A149E mutation could play a role in reducing DAP-induced delocalization of a MurAA–MurG complex *in vivo*. In wildtype *E. faecium*, MurAA localized principally at septa and poles, showing a clear colocalization pattern with the FM 4-64 FX stained membrane (Figs. 9 and S7). In P8 and P60, although MurAA^A149E^ was distributed both in the membrane and cytosol, the membrane-associated portion still showed a preference for the septa and poles (Fig. 9). Interestingly, membrane stain FM 4-64 FX showed clear indications of a speckled lipid distribution pattern, a phenotype commonly observed after *E. faecalis* adaptation to DAP (14).

**Figure 10:**
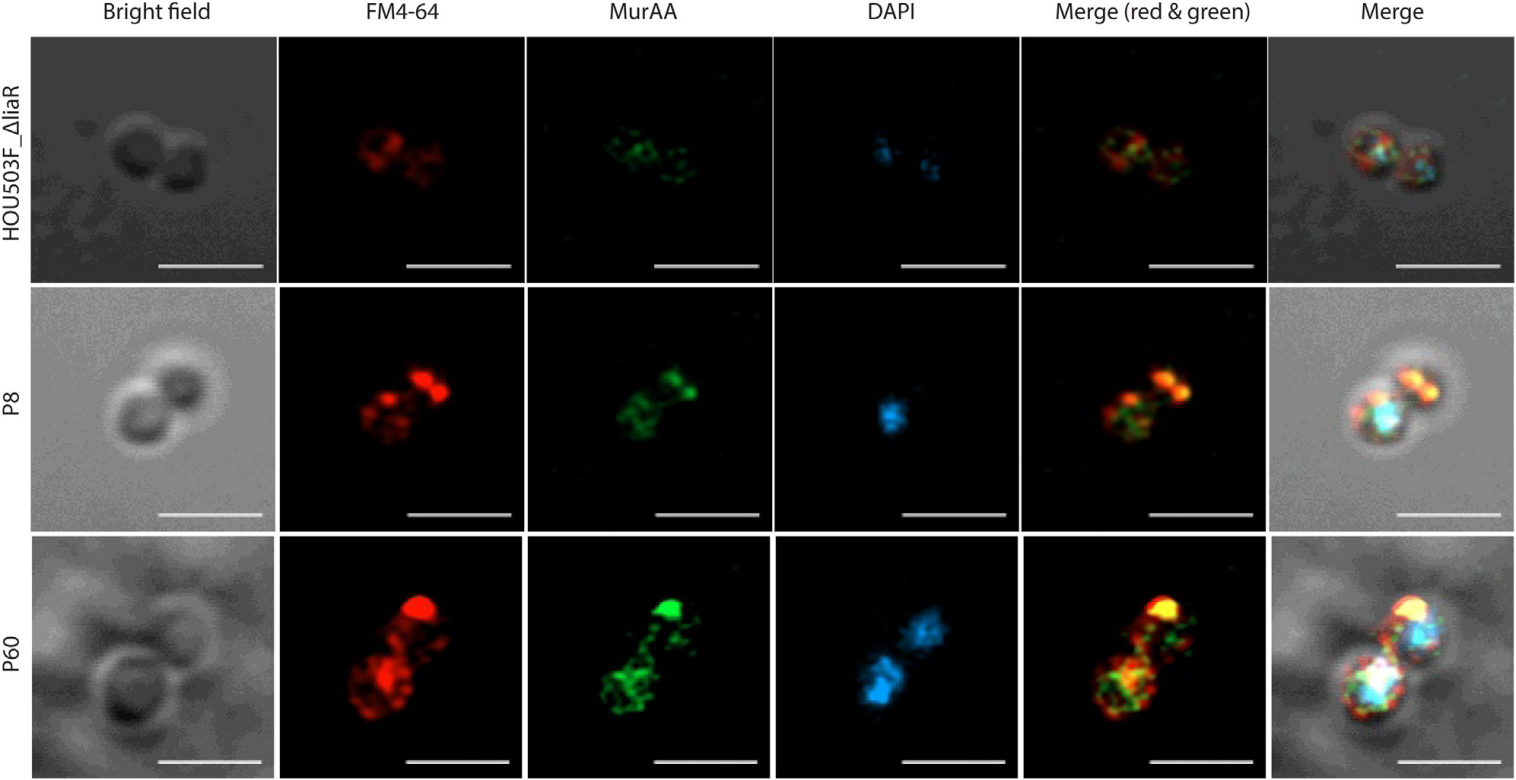
**MurAA was delocalized from membrane in HOU503F_Δ*liaR*, and MurAA**^**A149E**^**showed a modest decrease of association with the membrane in the two *murAA***^**A149E**^**isolates after DAP treatment.** Representative micrographs of *E. faecium* HOU503F_Δ*liaR* (ancestor), P8, and P60. Bacteria were grown to mid-log phase followed by treatment with 62.5 μg/l DAP for 10 min. Cells were stained with membrane dye FM 4-64 FX (*red*), DNA dye DAPI (*blue*), and MurAA antibody (*green*). *Top row* (HOU503F_Δ*liaR*): In the ancestor, DAP exposure led to a rapid and complete delocalization of MurAA from membrane to the cytosol. *Middle* and *bottom rows* (P8 and P60): Both P8 and P60 MurAA^A149E^ continued to show partial association with the damaged membrane at the poles and septa. The scale bar represents 2 μm. DAP, daptomycin.

**Supplemental Figure S5:**
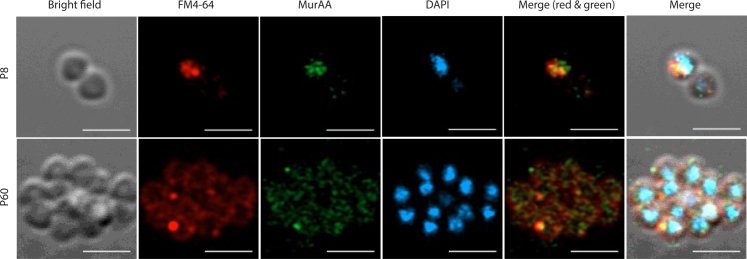


**Supplemental Figure S6:**
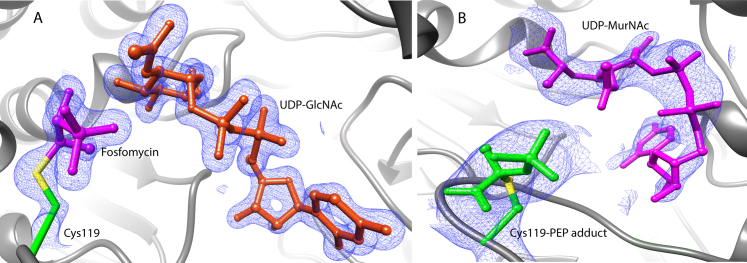


Exposure of the cells to a sub-MIC of DAP (62.5 μg/l, HOU503F_*ΔliaR* and 62.5 μg/l and 2 mg/l for P8 and P60) followed by lysozyme treatment to permeabilize the cells for staining showed clear indications of disrupted membrane integrity of both ancestor and *murAA*^A149E^ that significantly undermined sample quality due to increased lysis for P8 and P60. Interestingly, the DAP-exposed and lysozyme-permeabilized isolates showed strain-dependent effects on MurAA localization (Figs. 10, S5 and S7). In the ancestor, DAP exposure led to a significant (10-min treatment) dissociation of MurAA from the membrane to the cytosol, while the images for P8 and P60 suggested a more modest decrease of MurAA^A149E^ association with the damaged membrane at the poles and septa (Fig. S7). As P8 and P60 have a very high sensitivity to the lysozyme used to permeabilize cells for antibody staining, we were unable to increase DAP beyond 62.5 μg/l without significant membrane damage leading to cell lysis. Overall, MurAA^A149E^ P8 and P60 stayed well associated with the division septa during DAP exposure consistent with our *in vitro* studies showing a stronger association of MurAA^A149E^ with membrane-localized MurG.

## Discussion

Understanding the mechanistic basis for DAP resistance can provide important insights to the development of novel therapeutic approaches to treat recalcitrant, multidrug-resistant, and severe infections caused by *E. faecium* in vulnerable patients. The *liaFSR* envelope-stress-response pathway is commonly associated with DAP resistance in both *E. faecalis* and *E. faecium*, and it has been suggested that inhibition of the *liaFSR* pathway might be a strategy to restore DAP effectiveness or delay the development of resistance (21, 38). Previously, Prater *et al.* (18) showed that, in an *E. faecium* mutant lacking the gene encoding for the LiaR response regulator (hypersusceptible to DAP), alternative pathways for DAP resistance developed upon exposure to ascending concentrations of the antibiotic. Unexpectedly, in derivatives of *E. faecium* HOU503F_*ΔliaR*, resistance to DAP was accompanied by mutations in the gene encoding MurAA. In strains P8 and P60, MurAA^A149E^ was an important early adaptive change observed during the development of DAP resistance (21).

Because changes in the peptidoglycan synthesis pathways had not been observed previously, we performed comparative biochemical characterization of MurAA and MurAA^A149E^ to understand how changes in MurAA might provide increased protection during DAP exposure. Our results indicated that the A149E substitution only marginally affected the steady-state catalytic activity of the mutant enzyme compared with wildtype. We also noted a modest 2- to 3-fold increase in expression of *murAA* and *murAB* suggesting that increased expression of *murAA* alleles may help compensate for the modestly reduced activity, and also suggesting that changes in enzymatic activity alone were not likely to be the major drivers in the development of DAP resistance in strains P8 and P60 that express MurAA^A149E^. Based on the structure of *E. faecium* MurAA, both the A149E and G220V mutations are located in the catalytic domain of the protein but distal from the active site (Fig. 1). In addition to being quite distant from the active site, positions 149 and 220 are proximal to each other, and are both surface exposed, suggesting that these mutations could comprise part of a protein surface involved in interactions with other proteins.

Earlier studies in other organisms have identified interactions between MurAA and MurG, and therefore, we determined whether the MurAA^A149E^ substitution might affect a potential interaction in *E. faecium*. Indeed, our studies showed that the MurAA^A149E^–MurG complex was ∼16-fold stronger than that formed with the wildtype enzyme. Taken together, the strong change in binding affinity for MurG, the positions of the adaptive changes (at the protein surface and away from the active site), coupled with only modest changes in enzyme activity, suggest that adaptive changes in MurAA may improve the ability of *E. faecium* to withstand the cell envelope damage caused by DAP *via* changes in protein–protein interactions in critical peptidoglycan synthesis enzymes.

In *E. coli*, MurG is localized in the lateral cell wall and sites of division (39). In *B. subtilis*, MurG–MreB are preferentially bound to regions of increased fluidity (40), which are also targeted by DAP (10). In *Caulobacter crescentus*, MurG localizes in a FtsZ-dependent manner to division septa where peptidoglycan is actively synthesized (22). Since we identified a strong interaction of *E. faecium* MurAA–MurG *in vitro*, we hypothesized that MurAA localizes to the division septa in association with MurG *in vivo*. Using fluorescence microscopy, we observed that MurAA appeared to be associated with the cell membrane and strongly at the septa in cells isolated during exponential growth.

MurG is responsible for the final cytosolic step in the production of peptidoglycan as it converts lipid I into lipid II. Accumulation of MurG and other members of the Mur family of enzymes at the septa can increase enzymatic efficiency and localize peptidoglycan production where it is needed. In the absence of DAP-induced stress, immunofluorescence studies of the two strains expressing MurAA^A149E^ showed evidence for MurAA in both the cytoplasm and septa. Upon addition of DAP, variants expressing MurAA^A149E^ were better retained at the division septa when compared with wildtype. We speculate that the MurAA^A149E^–MurG complex concentrated at the septum may provide a sufficient supply of lipid II for peptidoglycan synthesis during cell division during DAP exposure. It was interesting that both strains expressing MurAA^A149E^ (P8 and P60) had markedly increased sensitivity to mutanolysin and lysozyme. It may be that there is a trade-off in which the increased stability of the MurAA–MurG complex comes at the cost of increased overall sensitivity to glycoside hydrolases. If so, the expression of MurAA^A149E^ in cells may render them more sensitive to cell wall hydrolases *in vivo*, making them potentially more susceptible to the host innate immune response.

Membrane modifications are also important drivers for DAP resistance. The subsequent mutations such as those in *cls* were commonly observed in DAP-resistant enterococci, and this supports the importance of changes in membrane phospholipids to improve cellular fitness during DAP exposure. In *E. coli*, cardiolipin copurifies with MurG (41), and in *B. subtilis*, cardiolipin is necessary to localize MurG to the forespore (23). We have shown previously that adaptive mutations in *cls* to produce Cls^H215R^ and Cls^R218Q^ moderately increase Cls activity (42). The observed mutations of *cls* in P8 and P60 may be another factor affecting the MurG localization under DAP exposure. Changes in Cls are consistent with adaptive changes in anionic phospholipid microdomains that could serve as a mechanism to further localize proteins involved in cell division.

In Prater *et al.* 2021 (21), we showed that deletion of *liaR* from the genome of *E. faecium* favored the evolution of diverse strategies to DAP resistance. The increased affinity of MurAA for MurG after acquiring the A149E mutation suggests that the change at position 149 affects the interaction of MurAA with its partner. It is also possible that MurAA has other partners and serves as the scaffold to recruit proteins related to cell wall synthesis. The mutation we identified could affect the stability of the entire complex, further affecting the cellular fitness during DAP exposure. The finding of MurAA–MurG localization at the septa together with the decreased delocalization imparted by MurAA^A149E^ provides a new perspective on the relationship of peptidoglycan homeostasis and the role of adaptive mutations in mitigating DAP-induced mislocalization of essential protein complexes.

## Experimental procedures

### Strains and growth conditions

Clinical isolate *E. faecium* HOU503 with deletions of liaR encoding the response regulator of the LiaFSR system were used (denoted as HOU503F_*ΔliaR*). Initial DAP MIC in Brain Heart Infusion (BHI) was 0.25 mg/l.

### Plasmid construction

The gene encoding full-length MurAA^WT^ was amplified by PCR from *E. faecium* HOU503F_*ΔliaR*, cloned into a modified 6∗His-SUMO tag–fused pETDuet vector by Gibson assembly and expressed in *E. coli* BL21 (DE3) cells using LB medium. Plasmid for the expression of *E. faecium* MurAA^A149E^ was generated by site-directed mutagenesis. The gene encoding full-length MurG was amplified by PCR from *E. faecium* HOU503F_*ΔliaR*, cloned into pETDuet vector by Gibson assembly, and expressed in *E. coli* BL21 (DE3) cells using LB medium (see Table S3).

### Expression and purification of *E. faecium* MurAA^WT^/MurAA^A149E^ and MurG

*E. coli* BL21 (DE3) was grown in LB medium supplemented with 100 μg/ml ampicillin at 37 °C until absorbance reached 0.55 to 0.65. Isopropyl-β-d-thiogalactopyranoside (IPTG), 0.4 mM, was added, and cells were grown at 16 °C for another 20 h before harvesting by centrifugation. MurAA: Cells were suspended in the lysis buffer (50 mM Tris pH 7.5, 1 M NaCl, 10% Glycerol [v/v], 20 mM imidazole, 1 mM dithiothreitol [DTT], 0.2 mM phenylmethylsulfonyl fluoride) and lysed by sonication. Supernatants were collected by centrifugation at 24,000 rpm at 4 °C for 40 min. Protein was purified with gravity flow chromatography column packed with Ni-NTA agarose resin and step eluted with buffer containing 100 to 500 mM imidazole. Collected fractions containing target protein were pooled and dialyzed overnight at 4 °C against 50 mM Tris pH 7.5, 500 mM NaCl, 10% Glycerol (v/v), and 1 mM DTT. The N-terminal 6∗His-SUMO tag was removed with His-tagged SUMO protease. The plasmid encoding SUMO protease ULP1 was transformed into Rosetta (DE3) competent cells. The SUMO protease was purified using a gravity Ni-NTA column. The untagged protein was further purified using a 5-ml HisTrap nickel affinity column (GE Healthcare) and gradient eluted with buffer containing 50 mM Tris pH 7.5, 500 mM NaCl, 10% Glycerol (v/v), 500 mM imidazole, and 1 mM DTT. The fractions containing protein were collected and further purified by Superdex 200 gel filtration chromatography (GE Healthcare) in the buffer containing 50 mM Tris pH 7.5, 225 mM NaCl, and 5% Glycerol (v/v).

MurG: Cells were suspended in the lysis buffer (20 mM Hepes pH 7.5, 500 mM NaCl, 10% glycerol [v/v], 20 mM Imidazole, 3% Triton X-100 [v/v], 1 mM DTT, and a tablet of cocktail protease inhibitor [Roche cOmplete, Mini, EDTA-free Protease Inhibitor Cocktail]) and lysed by sonication. Supernatants were collected and purified through nickel affinity column followed by size-exclusion chromatography in the buffer containing 20 mM Hepes pH 7.5, 500 mM NaCl, 10% glycerol (v/v), and 2 mM DTT.

### Structure determination of full-length MurAA

Purified protein was stored in buffer containing 50 mM Tris pH 7.5, 225 mM NaCl, and 5% Glycerol (v/v). MurAA crystals used for data collection were grown in two conditions: (1) 10 mg/ml protein in the presence of 2.5 mM UDP-GlcNAc and 2.5 mM FOS in the buffer and then mixed in a 1:1 ratio with the reservoir solution containing 0.22 M Potassium sodium tartrate tetrahydrate, 26% w/v Polyethylene glycol 3350, pH 7.4. The crystals were grown at 20 °C using the hanging drop vapor diffusion method. (2) Protein, 15 mg/ml, mixed in a 1:1 ratio with the reservoir solution containing 2.1 M DL-Malic acid pH 7.0 at 20 °C using the sitting drop vapor diffusion method. Crystals were cryoprotected in a solution consisting of the reservoir buffer supplemented with 20% (v/v) glycerol. Diffraction data were recorded at a synchrotron radiation wavelength of 0.98 Å at 100 K on 21-ID-G beamline at the Advanced Photon Source, Argonne, Illinois, using a charge coupled device detector. For the first condition: Crystals diffracted at a maximum resolution of 1.65 Å, and diffraction data were indexed and scaled by using the XDS package (43). Further processing was done by using the AIMLESS program (44) of the CCP4 suite (45). Indexing and analysis of systematic absences indicated that the crystals belonged to the P1 space group. The structure was determined by molecular replacement method using Phaser (46) with the template model generated from *Listeria monocytogenes* MurA (PDB id: 3R38) by excluding all solvent atoms. The structure was refined by several cycles of rigid body refinement followed by iterative cycles of restrained refinement using Refmac5 of CCP4 suite (45, 47). We tested both isotropic and anisotropic temperature factor refinement and observed better results when refining the B factor anisotropically as indicated by R and Rfree factors. The structure refinement was followed by multiple cycles of manual model rebuilding using COOT (48). Interpretable electron density was observed for all the amino acid residues of MurAA. The initial difference Fourier electron density map showed unambiguous electron density near the active site, which was interpreted and modeled as UDP-GlcNAc and FOS. The final refined structure was validated for quality and stereochemistry by using Molprobity (49). The structure-depicting figures were made using PyMOL (http://www.pymol.org/pymol) and UCSF chimera (50) programs. Final refined coordinates and experimental phases for MurAA ternary complex with UDP-GlcNAc and FOS were deposited in PDB with accession number 7TB0. For the second condition: The diffraction data were processed using XDS (43), and phases were calculated by phaser program (46) using the structure of a single polypeptide chain of MurAA–PEP–UDP–MurNAc structure after removing ligands and solvent molecules as a template. The structure was refined by using refmac5 (47) by rigid body refinement followed by iterative cycles of restrained refinement and model building in COOT (48). The structure was deposited in PDB with accession number 8D84.

### Kinetics measurement of MurAA activity

The manufacturer’s protocol was adapted and optimized (EnzChek Phosphate Assay Kit [E-6646]) as follows. To measure the values of the kinetic parameters for wildtype and mutant of MurAA against PEP, 125 μl of reaction mixture containing 35 nM of MurAA^WT^/MurAA^A149E^ and 4 mM UDP-GlcNAc was incubated with six concentrations (5, 10, 15, 25, 50, 75 μM) of PEP in Corning 96-well half-area microplates (CLS3695). Other components in the reaction mixture (reaction buffer, 2-amino-6-mercapto-7-methylpurine riboside, purine nucleoside phosphorylase) were provided in the kit and used following the manufacturer’s protocol. The mixture was preincubated at 22 °C for 10 min before initiating the reaction by adding PEP. The absorbance at 360 nm was immediately recorded for 40 min at intervals of 20 s. To measure the Km of UDP-GlcNAc, eight concentrations of UDP-GlcNAc (10, 20, 30, 40, 50, 100, 250, 500 μM) were used with saturating amount of PEP (1 mM) and 50 nM of MurAA. The reaction was initiated by adding the UDP-GlcNAc to the MurAA–PEP reaction system. Initial velocity was calculated by taking the linear portion of the enzyme progression curve, and the Vmax and Km were calculated by plotting the data into the Michaelis–Menten equation using GraphPad. The assays were performed in triplicate.

### Cys119 reactivity measurements using microscale thermophoresis

The reactivity of the active site Cys119 of MurAA^WT^/MurAA^A149E^ for FOS was measured by microscale thermophoresis using a Monolith NT.115 system (Nanotemper Technologies). MurAA was fluorescently labeled by mixing 100 μl of 200 nM MurAA and 100 μl of 100 nM dye provided in the kit followed by incubation for 30 min. Fifty nanomoles fluorescently labeled MurAA^WT^/MurAA^A149E^ and 2 nM UDP-GlcNAc mixture was incubated with a serial dilution of FOS in the reaction buffer (20 mM Hepes pH 7.5, 150 mM NaCl, 0.05% Tween). The reaction proceeded in darkness for 30 min, and samples were loaded into standard treated capillaries (Nanotemper). The measurements were performed in Nano-RED mode with an excitation power of 40%. Data were analyzed by Nanotemper MO.Affinity analysis software v2.3. Experiments were performed in triplicate.

MurAA–MurG affinity measurements using MST. To measure the MurAA–MurG binding affinity, purified His-MurG was labeled using a His-Tag Labeling Kit RED-tris-NTA 2nd Generation (Nanotemper MO-L018) and following the manufacturer’s protocol. MurG was incubated for 30 min in the dark at room temperature. 50 nM Labeled MurG, 50 nM, was mixed with a serial dilution of MurAA^WT^ (from 402 μM to 12.3 nM) or MurAA^A149E^ (from 69.9 μM to 2.13 nM) in the reaction buffer (20 mM Hepes pH 7.5, 150 mM NaCl, 0.05% Tween). The mixture was loaded into Monolith NT.115 Capillaries (MO-K022), and measurement was performed with Monolith NT.115 instrument. Data were analyzed by MO.Affinity Analysis v2.3. Experiments were performed in triplicate.

### Secondary structure motifs and thermostability comparison by circular dichroism

Secondary structure and thermal stability of MurAA^WT^/MurAA^A149E^ were measured by Jasco J 815 Circular Dichroism Spectrometer. For estimations of secondary structure, the CD spectrum was measured from 260 nm to 190 nm at 3 μM protein in a 2-mm-path-length cuvette. For thermostability, measurements were performed at an interval of 0.1 °C for every 5 s using a wavelength of 220 nm. Experiments were performed in triplicate.

### Reverse transcription–quantitative polymerase chain reaction

Overnight cultures of *E. faecium* were reinoculated into 5 ml fresh BHI and harvested at *A*_600_ of 0.5. Cells were incubated with 83.33 U/ml mutanolysin and 8.33 mg/ml lysozyme at 37 °C for 30 min prior to RNA extraction using RNeasy Mini Kit (Qiagen 74104). Contaminating DNA was removed using DNase I and checked by PCR. cDNAs were synthesized using Invitrogen SuperScript III, and RT-qPCR was performed with iQ SYBR green Supermix (BioRad 1708880) using the Bio-Rad CFX connect real-time PCR instrument. *gdhIV* was used as the housekeeping gene. Experiments were performed in biological and technical triplicate.

### Mutanolysin and lysozyme sensitivity assay

Overnight cultures normalized to an *A*_600_ of 0.05 were inoculated into fresh BHI supplemented with eight concentrations of mutanolysin and lysozyme in 96-well plates (mutanolysin: 0, 6.25, 12.5, 25, 50, 75, 100, 200 U/ml; lysozyme: 0, 1.25, 2.5, 5. 10, 15, 20, 40 mg/ml). Absorbances at *A*_600_ were measured every 5 min for 24 h using a plate reader (Corning Incorporated costar REF 3595).

### Dot blot assay

Different amounts of purified MurG (from 0.63 ng to 0.66 μg), bovine serum albumin (BSA), and MurAA^WT^/MurAA^A149E^ were spotted onto the nitrocellulose membrane in a volume of 2 μl. Negative control (BSA) was loaded at 1 μg, and positive controls were loaded at 0.66, 1, and 2 μg. The membrane was dried for 30 min followed by blocking with 1% BSA for 1 h at room temperature. After washing in PBS-Tween 0.05% (PBS-T), the membrane was incubated in 0.1 mg/ml MurAA^WT^/MurAA^A149E^ overnight at 4 °C. The membrane was incubated with anti-MurAA primary antibody (HRP-Conjugated Polyclonal Antibody raised in rabbit from GenScript) at a dilution of 1:500 (1.2 μg/ml) overnight at 4 °C. Detection was performed with SIGMAFAST DAB tablets with Metal Enhancer. Experiments were performed in duplicate.

### Immunofluorescence microscopy

*E. faecium* 503FΔ*liaR* (DAP-sensitive strain) and two DAP adaptive strains P8 and P60 were grown in BHI at 37 °C until mid-log phase. The experimental groups were treated with 50 mg/l calcium chloride and various concentrations of DAP for 10 min. 503FΔ*liaR*: 62.5 μg/l (one-fourth of 503FΔ*liaR* DAP MIC in BHI). P8: 62.5 μg/l and 2 mg/l (one-fourth of P8 DAP MIC in BHI). P60: 62.5 μg/l and 2 mg/l (one-fourth of P60 DAP MIC in BHI). The control groups were left untreated. Cells were harvested by centrifugation followed by three washes with PBS. Pellets were incubated with 2 μg/ml FM 4-64 FX on ice in the dark for 5 min followed by PBS wash. Cells were fixed with 4% preheated paraformaldehyde at room temperature for 30 min and washed three times in PBS. A volume of 50 μl of resuspended cells was immobilized on poly-L-lysine coated coverslip for 1 h, and unbound cells were washed out with PBS. Attached cells were permeabilized with various concentrations of mutanolysin/lysozyme pair at 37 °C for 1 h followed by 0.1% Triton in PBS treatment. Ancestor: 12.5 mU/ml mutanolysin + 1.25 μg/ml lysozyme. P8: 6.25 mU/ml mutanolysin + 0.63 μg/ml lysozyme for control groups and 3.13 mU/ml mutanolysin + 0.31 μg/ml lysozyme for experimental groups. P60: 1.56 mU/ml mutanolysin + 0.16 μg/ml lysozyme for control groups and no mutanolysin/lysozyme treatment for experimental groups. Cells were blocked with 10% BSA at room temperature for 1 h and incubated with primary antibody overnight at 4 °C. Slides were blocked again with 10% BSA for 1 h followed by secondary anti-rat Alexa 488 conjugate antibodies treatment (Molecular Probes, Thermo Fisher). Unbound probes were removed by thoroughly washing with PBS. Slides were incubated with 1 μg/ml DAPI at room temperature in the dark for 5 min. ProLong Gold, 10 μl, was added and slides were sealed using nail polish.

Super-resolution imaging of the samples was performed using a confocal laser scanning microscope Zeiss LSM800 (Carl Zeiss AG) equipped with a 32-concentric GaAsP Airyscan Super-Resolution (AS-SR) detector. To maximize the resolution enhancement, we used the dedicated Plan-Apochromat 63×/1.40 Oil objectives (Carl Zeiss) and Immersol 518 F 23 °C oil immersion (Carl Zeiss). Images were captured using Zen 2.6 - Blue edition (Carl Zeiss). Frame size was set using Optimal mode at 2586 × 2586 pixels with pixel size 0.06 μm. For our fluorophores (DAPI, Alexa Fluor 488, and FM4-64), the following acquisition settings were used: DAPI using 405 nm laser line with emission filter 400 nm to 475 nm, Alexa Fluor 488 using 488 nm laser line with emission filter 497 nm to 606 nm, and FM4-64 using 561 nm laser line with emission filter 564 nm to 700 nm. Image averaging was not applied. After raw data were captured, they were then processed using Airyscan Processing module available in Zen 2.6 - Blue edition (Carl Zeiss) with 2D SR processing option, and the Airyscan filtering (Wiener filter associated with deconvolution) was set to Standard.

## Data availability

Structures have been deposited in the RCSB Protein Data Bank (PDB) as accession numbers 7TB0 and 8D84. All remaining data are found in the article or supporting information.

## Supporting information

This article contains supporting information.

## Conflict of interest

C. A. A. has received grants from Merck, MeMEd Diagnostics, and Entasis Therapeutics. T. T. T. has received a grant from Merck.

## Figures and Tables

**Supplemental Figure S7:**