# Supplementary material for: Enolpyruvate transferase MurAAA149E, identified during adaptation of Enterococcus faecium to daptomycin, increases stability of MurAA–MurG interaction
Source: J Biol Chem. 2023 Jan 14;299(3):102912. doi: 10.1016/j.jbc.2023.102912 (PMC9975281; doi:10.1016/j.jbc.2023.102912)
Supplement: Supplemental Tables S1–S3 [file mmc1.docx]

**Table S1. Summary of data collection and structure refinement of *E. faecium* MurAA-FOS-** **UDP-GlcNAc**

|  | ***E. faecium* MurAA-FOS-** **UDP-GlcNAc** |
| --- | --- |
| Wavelength (Å) | 0.98 |
| Resolution range (Å) | 47.77 - 1.65 (1.71 - 1.65) |
| Space group | P 1 |
| Unit cell |  |
| a, b, c (Å) | 72.03 96.81 96.82 |
| α, β, γ (°) | 115.06 99.15 103.88 |
| Total reflections | 1024781 (100965) |
| Unique reflections | 257218 (25318) |
| Multiplicity | 4.0 (4.0) |
| Completeness (%) | 97.27 (95.68) |
| Mean I/sigma(I) | 9.09 (1.94) |
|  |  |
| R-merge | 0.101 (0.828) |
| R-meas | 0.1167 (0.956) |
| R-pim | 0.05847 (0.479) |
| CC1/2 | 0.995 (0.635) |
| CC* | 0.999 (0.881) |
|  |  |
|  |  |
| R-work (%) | 13.50 (23.78) |
| R-free (%) | 17.22 (26.85) |
|  |  |
|  |  |
| Number of non-hydrogen atoms | 14654 |
| macromolecules | 12853 |
| ligands | 205 |
| solvent | 1596 |
| Protein residues | 1680 |
| RMS(bonds) (Å) | 0.016 |
| RMS(angles) (°) | 1.79 |
| Ramachandran favored (%) | 98.03 |
| Ramachandran allowed (%) | 1.79 |
| Ramachandran outliers (%) | 0.18 |
|  |  |
|  |  |
| Average B-factor (Å2) | 20.36 |
| macromolecules | 18.93 |
| ligands | 14.42 |
| solvent | 32.62 |

*Statistics for the highest-resolution shell are shown in parentheses.

**Table S2. Summary of data collection and structure refinement of *E. faecium* MurAA-PEP-**

**UDP-MurNAc**

|  | ***E. faecium* MurAA-PEP- UDP-MurNAc** |
| --- | --- |
| Wavelength (Å) | 0.98 |
| Resolution range (Å) | 49.76 - 2.65 (2.75 - 2.65) |
| Space group | P 2 21 21 |
| Unit cell |  |
| a, b, c (Å) | 146.88 168.39 316.19 |
| α, β, γ (°) | 90 90 90 |
| Total reflections | 3399385 (321678) |
| Unique reflections | 225455 (22047) |
| Multiplicity | 15.1 (14.6) |
| Completeness (%) | 99.78 (98.20) |
| Mean I/sigma(I) | 11.32 (0.93) |
|  |  |
| R-merge | 0.2363 (3.06) |
| R-meas | 0.2446 (3.17) |
| R-pim | 0.06295 (0.825) |
| CC1/2 | 0.997 (0.368) |
| CC* | 0.999 (0.733) |
|  |  |
|  |  |
| R-work (%) | 25.39 (37.73) |
| R-free (%) | 29.64 (39.53) |
|  |  |
|  |  |
| Number of non-hydrogen atoms | 39068 |
| macromolecules | 38501 |
| ligands | 528 |
| solvent | 39 |
| Protein residues | 5039 |
| RMS(bonds) (Å) | 0.020 |
| RMS(angles) (°) | 2.01 |
| Ramachandran favored (%) | 97.09 |
| Ramachandran allowed (%) | 2.77 |
| Ramachandran outliers (%) | 0.14 |
|  |  |
|  |  |
| Average B-factor (Å2) | 68.79 |
| macromolecules | 67.74 |
| ligands | 146.53 |
| solvent | 49.96 |

*Statistics for the highest-resolution shell are shown in parentheses.

**Table S3. Primers used in this study**

| Primer | Sequence |
| --- | --- |
| *E. faecium* MurAA forward primer | CAGATTGGTGGTCATATGATGGAAGAGATCATCGTAAGAGGTGG |
| *E. faecium* MurAA reverse primer | CTTTACCAGACTCGAGTTAAGCAATCGTTTGTGCTGTTTTTTCAGC |
| *E. faecium* MurAA forward primer for pETDuet | CGATTGCTTAACTCGAGTCTGGTAAAGAAACCGCT |
| *E. faecium* MurAA reverse primer for pETDuet | CTCTTCCATCATATGACCACCAATCTGTTCTCTGTG |
| *E. faecium* MurG forward primer | CGAGAACCTGTACTTCCAATCCATGAAAATTTTGGTTACAGGCGG |
| *E. faecium* MurG reverse primer | ACCAGACTCGAGTAAGACCACCGCCTTTATTTTTTG |
| *E. faecium* MurG forward primer for pETDuet | AGGCGGTGGTCTTACTCGAGTCTGGTAAAGAAACC |
| *E. faecium* MurG reverse primer for pETDuet | ATGGATTGGAAGTACAGGTTCTCGTGATGATGATGATGATGGCTG |
