# Supplementary material for: Enolpyruvate transferase MurAAA149E, identified during adaptation of Enterococcus faecium to daptomycin, increases stability of MurAA–MurG interaction
Source: J Biol Chem. 2023 Jan 14;299(3):102912. doi: 10.1016/j.jbc.2023.102912 (PMC9975281; doi:10.1016/j.jbc.2023.102912)
Supplement: Supplemental Figure Legends [file mmc2.docx]

**Supplementary Information**

**Enolpyruvate Transferase MurAA^A149E^, Identified During Adaptation of *Enterococcus faecium* to Daptomycin, Increases Stability of MurAA-MurG Interactions Authors**

Dr. Yue Zhou^1^, Dr. Budi Utama^2^, Dr. Shivendra Pratap^1^, Adeline Supandy^1^, Xinhao Song^1^, Dr. Truc T. Tran^3^, Dr. Heer H. Mehta^1^, Dr. Cesar A. Arias^3,4^, Dr. Yousif Shamoo^1^

**Affiliations**

^1^Department of Biosciences, Rice University, Houston, Texas, USA.

^2^Shared Equipment Authority, Rice University, Houston, Texas, USA.

^3^Center for Infectious Diseases Research, Houston Methodist Research Institute, Houston, Texas, USA.

^4^Division of Infectious Diseases, Houston Methodist Hospital, Houston, Texas, USA.

**Figure S1.** **A149E mutation at the surface loop is 24.7 Å away from the Cys-119.** MurAA is shown in green. Active site Cys-119 is in magenta and A149E mutation is in red. FOS and UDP-GlcNAc are shown as sticks.

**Thermal denaturation and secondary structure are not strongly affected for MurAA^A149E^ mutant compared to wild type.** The secondary structure and thermal stability of *E. faecium* MurAA^WT^ and MurAA^A149E^ were measured to estimate the effect of A149E mutation on protein folding. The overall curves of the two proteins are quite similar (Figure S2 A). The deep well shape appearing from 230 to 210 nm indicates a strong alpha helix motif, consistent with the structure. The well-defined motif and similar circular dichroism (CD) curve suggest both MurAA^WT^ and MurAA^A149E^ are correctly folded and their overall structures were likely to be similar. Thermal stability of the protein was investigated by measuring the melting temperature *Tm* of MurAA^WT^ and MurAA^A149E^. The result indicates that *Tm* is roughly the same between two proteins, where MurAA^WT^ has a *Tm* value of 54.92 ± 0.09 °C and MurAA^A149E^ has a value of 54.04 ± 0.15 °C (Figure S2 BC). Thus, our data indicate that A149E mutation doesn’t interfere with protein folding.

**Figure S2.** **Neither secondary structure nor thermal stability of *E. faecium* MurAA change dramatically after acquiring A149E mutation.**

(A) Circular dichroism spectra indicate *E.faecium* MurAA^WT^ (blue) and MurAA^A149E^ (red) are well folded. Molar ellipticity is the absorbance difference corrected for sample concentration. (B) (C) Thermal stability of *E. faecium* MurAA^WT^ and MurAA^A149E^. Melting temperature of MurAA^WT^ (blue) is 54.92 ± 0.09 °C and that of MurAA^A149E^ (red) is 54.04 ± 0.15 °C.

**Figure S3. MurAA^WT^ is mostly soluble whereas MurAA^A149E^ is mostly insoluble.** The thick band in supernatant versus the thin band in pellet indicates MurAA^WT^ is largely soluble, and it is opposite for MurAA^A149E^ which indicates most of the expressed protein is insoluble. The protein was induced under 16 °C for 20 h with 0.4 mM IPTG. S: supernatant. P: pellet. The unit of molecular weight marker is kDa.

**Figure S4. Dot blot assay indicated that MurG interacts with MurAA^WT^** **and MurAA^A149E^.** (A) Bottom row from right to left were positive controls MurAA^WT^, negative control BSA and MurG. Top row from right to left was a serial dilution of MurG ranging from 0.33 μg to 0.63 ng. (B) Bottom row from right to left were positive controls MurAA^A149E^, negative control BSA and MurG. Top row from right to left was a serial dilution of MurG ranging from 0.33 μg to 0.63 ng.

**Figure S5. High doses of DAP disrupted membrane integrity and delocalized the MurAA from the membrane.** In the two murAA^A149E^ isolates of *E. faecium* P8 (upper panel) and P60 (lower panel) stained with membrane dye FM™ 4-64 FX (red), DNA dye DAPI (blue) and MurAA antibody (green) after treatment with 2 mg/L DAP for 10 min. Compared to Fig. 9 and 10, the high dose DAP treatment shown in this figure significantly lowered the yellow color level of co-localization between FM™ 4-64 FX (red) and MurAA antibody (green) in membrane regions. Scale bars 2 µm.

**Electron density from *E. faecium* MurAA (8D84) was fitted to UNAM but was variable across different copies of the protein in the asymmetric unit.** The variable quality of the electron density in these regions suggests partial occupancy of UNAM. As UNAM was not included in the original crystallization condition, we speculate that UNAM was co-purified with MurAA.

**Figure S6:** Electron density of complexes. A. Covalent adduct of active site Cys119 with Fosfomycin along with adjacently bound UDP-GlcNAc in the MurAA-FOS- UDP-GlcNAc complex structure (PDB: 7TB0). B. Covalent adduct of active site Cys119 with PEP along with bound UDP-MurNAc in MurAA-PEP- UDP-MurNAc structure (PDB: 8D84). The density maps (Blue) are made from a 2Fo - Fc Fourier synthesis and are contoured at σ level of 1. Electron density is overlaid on the structure model (ball and stick). Active site Cys119 and PEP are colored in Green, Fosfomycin and UDP-MurNAc are colored in Magenta, UDP-GlcNAc is shown in Orange.

**P8 and P60 have higher sensitivity to mutanolysin and lysozyme permeabilization treatment.** In the immunofluorescence microscopy experiment, mutanolysin / lysozyme combination is used to permeabilize the cell wall. Generally, P8 and P60 are more sensitive to mutanolysin and lysozyme (also shown in fig 3). Compared to the ancestor, lower mutanolysin and lysozyme were added in two *murAA*^A149E^ isolates in order to keep the cell shape and not over lyse the cell.

**P8 and P60 had other mutations in their genome in addition to MurAA^A149E^**

*entfae_809, a* capsular exopolysaccharide family protein, is responsible for capsular polysaccharide biosynthesis and export (Geisinger and Isberg, 2015) and is associated with resistance to antimicrobial peptides (Campos et al., 2004) (Jones et al., 2009). *entfae_64* encodes an unknown protein and is located upstream of methionine sulfoxide reductase *msrA*, catalyzing the reduction of methionine-sulfoxide to methionine. We hypothesize that *entfae_64 may be a* regulator of *msrA* and the potential loss-of-function *entfae_64^Y83*^* mutation increase *msrA* expression. Other studies showed the bactericidal mechanism of DAP including overexpressing of reactive oxygen species (ROS) and inhibiting anti-ROS response (Po et al., 2021). *The* upregulation of msrA expression could combat the DAP-induced oxidative stress. *Entfae_126* encodes a conserved hypothetical protein and is located immediately upstream of the fusaric acid resistance protein-like family protein. The *entfae_126*^V30*^ may interfere with the transcription of the downstream fusaric acid resistance family protein. However, the reason for its appearance in DAP evolution remains unclear. Mutations in *cls* are commonly observed in DAP- resistant enterococci and are correlated with altered membrane dynamics and lipid composition (Tran et al., 2015b) (Supandy et al.).

**Preparation and co-localization analysis of permeabilized *E. faecium* HOU503ΔliaR*,* P8 and P60 strains by microscopy.** Figure S7 shows the extent of co-localization of the green fluorescence derived from the secondary antibody for the membrane. The P8 and P60 strain were very sensitive to the lysozyme used to permeabilize the cell wall leading to very significant lysis that undermined the quality of the samples. While we are confident that MurAA and MurAA^A149E^ associate with the membrane in their respective cell lines, we cannot infer that that the association of MurAA^A149E^ is stronger for MurG than wildtype as FM™ 4-64 FX is a membrane dye.

**Figure S7. Co-localization of MurAA (green) with membrane dye FM™ 4-64** **FX (red).** A) Co-localization of MurAA without DAP challenge. MurAA in the parental strain HOU503ΔLiaR, P8 and P60 were associated with the membrane though stain P8 showed a significantly lower association. B) When DAP is added to the cells prior to permeabilization, strains P8 and P60 (MurAA^A149E^) remained co-localized with the membrane to a moderately greater extent than HOU503ΔLiaR.
